# Phylogenetic farming: Can evolutionary history predict crop rotation via the soil microbiome?

**DOI:** 10.1111/eva.12956

**Published:** 2020-04-22

**Authors:** Ian Kaplan, Nicholas A. Bokulich, J. Gregory Caporaso, Laramy S. Enders, Wadih Ghanem, Kathryn S. Ingerslew

**Affiliations:** ^1^ Department of Entomology Purdue University West Lafayette IN USA; ^2^ Center for Applied Microbiome Science The Pathogen and Microbiome Institute Northern Arizona University Flagstaff AZ USA; ^3^ Department of Biological Sciences Northern Arizona University Flagstaff AZ USA

**Keywords:** crop rotation, phylogeny, plant–soil feedback, species relatedness, tomato

## Abstract

Agriculture has long employed phylogenetic rules whereby farmers are encouraged to rotate taxonomically unrelated plants in shared soil. Although this forms a central tenet of sustainable agriculture, strangely, this on‐farm “rule of thumb” has never been rigorously tested in a scientific framework. To experimentally evaluate the relationship between phylogenetic distance and crop performance, we used a plant–soil feedback approach whereby 35 crops and weeds varying in their relatedness to tomato (*Solanum lycopersicum*) were tested in a two‐year field experiment. We used community profiling of the bacteria and fungi to determine the extent to which soil microbes contribute to phenotypic differences in crop growth. Overall, tomato yield was ca. 15% lower in soil previously cultivated with tomato; yet, past the species level there was no effect of phylogenetic distance on crop performance. Soil microbial communities, on the other hand, were compositionally more similar between close plant relatives. Random forest regression predicted log_10_ phylogenetic distance to tomato with moderate accuracy (*R* ^2^ = .52), primarily driven by bacteria in the genus *Sphingobium*. These data indicate that, beyond avoiding conspecifics, evolutionary history contributes little to understanding plant–soil feedbacks in agricultural fields; however, microbial legacies can be predicted by species identity and relatedness.

## 1 INTRODUCTION

Sustainable farming methods often mimic patterns and processes that are characteristic of natural ecosystems with the assumption that unmanaged wild communities have undergone intense selection over evolutionary time, weeding out poor designs in favor of superior ones (Altieri, 1995; Denison, 2012). Thus, understanding how natural communities are structured and identifying the components that are disrupted by modern agricultural practices may offer novel insight on how to restructure cropping systems to enhance production (e.g., higher yield, greater water use efficiency, fewer inputs of pesticides, and/or fertilizer).

Perhaps the most dramatic difference between natural and agricultural systems lies in their varying levels of diversity. Even to the untrained eye, natural communities stand out in maintaining more plant species per unit area than crop fields. While richness and evenness are, historically, the two most popular means by which to quantify diversity, recent studies emphasize a more cryptic component: phylogenetic relatedness, defined as the amount of time since two species shared a common ancestor (Cavender‐Bares, Kozak, Fine, & Kembel, 2009; Vamosi, Heard, Vamosi, & Webb, 2009). Relatedness offers a quantitative estimate for the degree of shared evolutionary history, either between two co‐occurring individuals or averaged across an assemblage of species. This means that two communities can have identical richness, while differing drastically in relatedness. Spatial analyses of species coexistence in natural ecosystems report evidence for phylogenetic overdispersion, a pattern whereby locally assembled communities are more phylogenetically dissimilar than expected based on null models that randomly assemble communities without considering evolutionary history (Allan et al., 2013; Cavender‐Bares, Ackerly, Baum, & Bazzaz, 2004; Cooper, Rodríguez, & Purvis, 2008; Gerhold, Cahill, Winter, Bartish, & Prinzing, 2015). These findings imply that in agricultural systems configuring polycultures based on random crop arrangement is “unnatural” and may in fact be less successful than employing a targeted approach to maximize the phylogenetic distance separating crops.

Despite pleas for integrating a phylogenetic perspective on applied sciences (Ness, Rollinson, & Whitney, 2011) and insights that phylodiversity has offered in various applied disciplines (e.g., forest management, Jactel & Brockerhoff, 2007; habitat restoration, Verdú, Gómez‐Aparicio, & Valiente‐Banuet, 2012; ecosystem function, Srivastava, Cadotte, MacDonald, Marushia, & Mirotchnick, 2012; invasive species, Pearse & Altermatt, 2013; urban planning, MacIvor, Cadotte, Livingstone, Lundholm, & Yasui, 2016), the existing agricultural literature virtually ignores evolutionary history (but see Ingerslew & Kaplan, 2018; Miller & Menalled, 2015; Schellhorn & Sork, 1997). In some cases, crops may be taxonomically clustered, either intentionally or unintentionally, because of similar growing requirements (i.e., related species possess comparable tillage, fertility, and/or irrigation needs). This would be predicted if traits conferring adaptation to growth environments are phylogenetically conserved. An analogous process, called phylogenetic underdispersion, is sometimes observed in nature when related species co‐occur because they share traits allowing them to persist in unique or stressful environments (Forrestel, Donoghue, & Smith, 2014; Verdú & Pausas, 2007).

Most crop production guides, however, recommend avoiding consecutive plantings of related species (i.e., same genus or family) over time. Closely related species tend to be more ecologically similar, resulting in more intense competition for a limited pool of resources (Burns & Strauss, 2011; Losos, 2008). Seedling survival and growth, for example, are higher with increasing phylodiversity of neighboring vegetation (Castillo, Verdú, & Valiente‐Banuet, 2010; Webb, Gilbert, & Donoghue, 2006), presumably due to increasingly divergent abiotic requirements and ecomorphological traits associated with acquiring those resources (e.g., rooting depth). Thus, plants on average stand to benefit from associating with distantly related species via niche partitioning (Cadotte, 2013). A second major driver is evading consumers. Closely related plants are more likely to share parasitic insects and pathogens (Gilbert, Briggs, & Magarey, 2015; Gilbert & Webb, 2007; Novotny et al., 2006; Yguel et al., 2011). As a result, increasing phylodiversity is a good rule of thumb for improving the statistical likelihood of cultivating nonhosts in a crop field without underlying knowledge of pest biology or diet breadth. In the context of rotations, host‐specific, soil‐borne microbial pathogens are considered the primary driver of negative feedbacks from close relatives.

Existing evidence for soil‐mediated phylogenetic effects on plant performance and microbial communities are mixed and derive entirely from unmanaged systems. Phylogenetic influences on plant–soil feedbacks impacting plant growth are highly inconsistent across studies, likely depending on variables such as the amount of phylogenetic distance tested relative to the focal plant (Anacker, Klironomos, Maherali, Reinhart, & Strauss, 2014; Burns & Strauss, 2011; Dostál & Palečková, 2010; Fitzpatrick, Gehant, Kotanen, & Johnson, 2017; Kuťáková, Herben, & Münzbergová, 2018; Liu et al., 2012; Mehrabi, Bell, & Lewis, 2015; Mehrabi & Tuck, 2014; Münzbergová & Šurinová, 2015). Yet, phylogeny appears to play a relatively stronger role in structuring plant‐associated soil microbes; close relatives tend to share more similar communities of rhizosphere bacteria and fungi, particularly for plant pathogenic taxa (Barberan et al., 2015; Gilbert & Webb, 2007; Peay, Baraloto, & Fine, 2013; Sarmiento et al., 2017; Schroeder et al., 2019). In combining these two approaches, one study found that increasing phylogenetic distance between neighbors improved focal plant growth in field‐collected “live” soil, but after the soil was experimentally treated with fungicide the relationship dissipated (Liu et al., 2012). These data suggest that plant species‐ and/or genus‐specific fungal pathogens mediate the negative consequences of growing in the same soil as close relatives.

Surprisingly, no field studies have quantified the effects of plant phylogenetic diversity on crop yield and soil microbiomes in agriculture, despite the fact that agronomists widely advocate rotations based on these factors. In a recent greenhouse study, we found that relatedness did not predict the soil legacy of 36 crop and weed species on short‐term vegetative growth of potted tomato plants (Ingerslew & Kaplan, 2018). Here, we conducted a 2‐year field experiment using the same agricultural plant community to assess whether species relatedness impacts soil microbial legacies and tomato yield. In keeping with the central tenets of the phylogenetic diversity hypothesis, we predicted that plants more closely related to tomato imprint similar soil microbiomes, resulting in correspondingly lower yield, compared to more distantly related taxa.

## 2 MATERIALS AND METHODS

We conducted a 2‐year field experiment at the Meigs‐Throckmorton Purdue Agricultural Center near Lafayette, Indiana (USA), during 2017 and 2018. In year one, we conditioned the soil using 36 species of crops and agricultural weeds, varying widely in their relatedness to the focal crop, tomato. In year two, we cultivated tomato throughout the entire field to evaluate the legacy effect of the prior year plantings. The community of 36 species (22 crops, 14 weeds; Table 1) was selected to represent plants commonly encountered on diversified vegetable farms, both locally in our area and throughout much of the United States. Thus, all have the potential to generate a soil legacy in which tomato would subsequently grow. The species composition included solanaceous crops and weeds in the same genus or family as tomato (*Solanum lycopersicum*), as well as distant relatives (see Ingerslew & Kaplan, 2018 for phylogenetic tree illustrating the evolutionary relationships among plant taxa in this group).

**Table 1:** List of plant species used in study, along with taxonomic affiliation and domestication status. Species are organized in rank order from least (top) to most (bottom) closely related to the focal plant, tomato

Plant type	Family	Common name	Species name
Crop	Poaceae	Rye	*Secale cereale*
Crop	Poaceae	Wheat	*Triticum aestivum*
Crop	Poaceae	Barley	*Hordeum vulgare*
Crop	Poaceae	Corn	*Zea mays*
Crop	Poaceae	Oats	*Avena sativa*
Crop	Poaceae	Foxtail millet	*Setaria italica*
Wild	Poaceae	Crabgrass	*Digitaria sanguinalis*
Wild	Poaceae	Barnyard grass	*Echinochloa crus‐galli*
Wild	Cyperaceae	Yellow nutsedge	*Cyperus esculentus*
Crop	Brassicaceae	Collard greens	*Brassica oleracea*
Crop	Fabaceae	Bean	*Phaseolus vulgaris*
Crop	Fabaceae	Pea	*Pisum sativum*
Wild	Fabaceae	Red clover	*Trifolium pratense*
Wild	Malvaceae	Velvetleaf	*Abutilon theophrasti*
Crop	Cucurbitaceae	Watermelon	*Citrullus lanatus*
Crop	Cucurbitaceae	Pumpkin	*Cucurbita pepo*
Crop	Cucurbitaceae	Cucumber	*Cucumis sativus*
Crop	Amaranthaceae	Spinach	*Spinacia oleracea*
Wild	Amaranthaceae	Redroot pigweed	*Amaranthus retroflexus*
Wild	Amaranthaceae	Lambsquarters	*Chenopodium album*
Crop	Apiaceae	Carrot	*Daucus carota*
Crop	Asteraceae	Sunflower	*Helianthus annuus*
Crop	Asteraceae	Lettuce	*Lactuca sativa*
Wild	Asteraceae	Field thistle	*Cirsium discolor*
Wild	Asteraceae	Ragweed	*Ambrosia artemisiifolia*
Crop	Lamiaceae	Basil	*Ocimum basilicum*
Wild	Convolvulaceae	Morning glory	*Ipomoea pandurata*
Crop	Solanaceae	Tobacco	*Nicotiana tabacum*
Wild	Solanaceae	Jimsonweed	*Datura stramonium*
Crop	Solanaceae	Sweet pepper	*Capsicum annuum*
Crop	Solanaceae	Tomatillo	*Physalis philadelphica*
Wild	Solanaceae	Ground cherry	*Physalis pruinosa*
Crop	Solanaceae	Eggplant	*Solanum melongena*
Wild	Solanaceae	Horsenettle	*Solanum carolinense*
Wild	Solanaceae	Bittersweet nightshade	*Solanum dulcamara*
Crop	Solanaceae	Tomato	*Solanum lycopersicum*

This experiment was conducted in a single field (285 × 90 ft LW), which was previously (2016) planted in corn (var Becks 6175LL). Immediately before starting the experiment, in May 2017, we sampled soil (6‐inch depth) at four random locations in each of the eight replicated blocks used in the study. The four samples per block were mixed to provide one analytical sample, which was used to conduct a basic soil test as recommended for commercial growers (A&L Great Lakes Laboratories). Soil textural characterization was silty clay loam or clay loam (19% sand, 53% silt, 28% clay) with the following general characteristics: organic matter = 2.75, available phosphorus = 42.87 ppm, exchangeable potassium = 168 ppm, magnesium = 256.25 ppm, calcium = 1656.25 ppm, pH = 6.7, buffer pH = 7.0, cation exchange capacity = 11.95, and percent base saturation of cation elements: %K 3.65, %Mg 18.04, %Ca 69.4, and %H 8.91.

### 2.1 Soil conditioning

We cultivated 36 plant species in a randomized complete block design with 8 replicated blocks in a single field. We also included two plant‐free fallow control plots per block, resulting in 304 total plots (= 36 species + 2 controls × 8 blocks). A block consisted of two adjacent 285 ft length rows (between‐row spacing, 6 ft), with 19 plots equally split between the two rows. A plot was considered four consecutive plants of the same species in a row, with 3 ft between‐plant spacing, and a 6 ft buffer separating plot treatments. There was no space between neighboring blocks, that is, each two‐row block was immediately adjacent to the next. The field was tilled in late May 2017 before constructing raised beds covered in a double layer of black plastic mulch to reduce weed pressure with drip tape for irrigation. A preplanting fertilizer was added to the soil at the following rates: potash 0‐0‐60 (71 lbs/ac) and diammonium phosphate 18‐46‐0 (147 lbs/ac).

Seeds for each of the 36 species were germinated in the laboratory in the spring and fertilized weekly beginning 2 weeks after transplanting seedlings into pots in the greenhouse. See Ingerslew and Kaplan (2018) for details on germination procedures and seed sources. Because seedling size varied across species, we standardized germination times. On June 1, seedlings were transplanted into their randomly assigned field plots. Because pure species plot treatments were necessary for the experimental design, we applied the following herbicides between rows on July 7 and 31 to prevent natural weed infiltration: paraquat (Gramoxone SL 2.0) and S‐metolachlor (Dual II Magnum). Other pesticides (i.e., insecticides, fungicides) were not applied in either year of the study. We hand weeded within and between rows as needed throughout the growing season to maintain treatments.

Between October 9 and 18, all plants were harvested and removed from the field. To do so, we uprooted plants, removing the main taproot and as much of the larger roots as possible. On November 27, the herbicides glyphosate, sulfentrazone, and metribuzin were applied to the whole field to kill any remaining plants.

### 2.2 Tomato response

Because we aimed to measure tomato responses to soil legacy effects from year 1 species treatments, we grew tomatoes throughout the entire field with seedlings transplanted in the exact location where the previous year's plants grew. Some species were persistent in reestablishing from belowground rhizomes (e.g., thistle, horsenettle, some grasses); these plants were repeatedly pulled by hand as needed to avoid competing with tomato. On June 1, we transplanted 1,216 tomato seedlings (var RG 611) into the field (i.e., 304 plots × 4 plants per plot). Two weeks later, they were fertilized through the drip irrigation with a soluble fertilizer (30 gallons of 10‐34‐0 NPK).

During transplant, we collected soil from each plot for microbial and nutrient analyses to quantify the soil legacy from year 1 treatments. Bulk soil was collected rather than rhizosphere soil to isolate the species temporal legacy without the confounding influence of tomato conditioning, while also measuring the initial soil properties experienced by the roots of a new tomato seedling. To do so, we sampled from the top 3‐inch profile of the soil layer at each of the four locations in a plot where plants previously grew; then, we combined these samples, creating a single ca. 350 g soil sample per plot. Sterile nitrile gloves were used to avoid microbial contamination between plots. In the field, we temporarily stored samples in sealed plastic bags in a cooler, before placing them in a −20°C freezer in the laboratory until analysis. After manually homogenizing samples, a 2 g subsample was isolated for microbial analysis (see below sections). The remainder was sent to the University of Connecticut Soil Nutrient Analysis Laboratory (Storrs, Connecticut) where they were analyzed for plant‐available calcium, magnesium, phosphorus, potassium, sulfur, iron, manganese, copper, zinc, aluminum, and boron using a modified Morgan extractant.

Tomato harvest began August 22 when green fruits began ripening and lasted for 2 weeks. Blocks were harvested sequentially to avoid temporal effects on yield that may impact interpretation of treatments. Each plant was cut at its base, fruits were removed, and we measured total fruit yield per plant.

The program Phylomatic (Webb & Donoghue, 2005) was used to assign phylogenetic distances separating each species in the community from tomato. This continuous variable was used as a predictor to test the impact of relatedness on plant–soil feedbacks. To do so, average tomato fruit yield per plant was calculated for each plot as the response variable. Because some tomato seedlings died after transplant, we created averages based on the number of plants (out of 4 max.) remaining; most plots had 3–4 surviving to the end of the season for yield estimates. This allowed us to standardize data across plots, rather than using total plot yield, which assumes plant density is identical. One of the 36 species treatments—spinach, *Spinacia oleracea*—did not persist in year 1 and thus was removed from the analysis. The impact of phylogenetic distance on plant–soil feedback for tomato yield was tested using regression (Proc Reg in SAS v. 9.4). As a response variable, we used species means for the plant–soil feedback effect size, calculated as ln(species treatment/fallow control).

This was followed up with categorical tests (Proc GLM) comparing tomato yield: (i) fallow (1) versus plant treatments (35), (ii) tomato (self, 1 sp.) versus all other plants in the community (non‐self, 34 sp.), (iii) congener versus noncongener (i.e., *Solanum*, 4 sp. versus non‐*Solanum*, 31 sp.), and (iv) confamilial versus nonconfamilial (i.e., Solanaceae, 7 sp. versus non‐Solanaceae, 28 sp.). Comparison (i) was used to evaluate whether plants imprint legacies on the soil that are unique from bare ground. Comparisons (ii–iv) were used to determine whether phylogenetic threshold effects occur at the species, genus, or family levels, respectively.

To gauge potential nonmicrobial mechanisms underlying plant–soil feedbacks on tomato yield, we also compared tomato versus nontomato soil for each of the nutritional traits measured (Proc GLM). A Bonferroni correction was applied to the statistical outcome to account for the multiple univariate tests performed on each individual nutrient.

### 2.3 Amplicon library preparation, sequencing, and bioinformatics

A 250 mg soil subsample was analyzed by Argonne National Laboratory for community profiling of bacteria and fungi. Raw sequence data are accessible in the Qiita repository (ID 12546; Gonzalez et al., 2018).

#### 2.3.1 16S rRNA sequencing for bacterial community

Briefly, PCR amplicon libraries targeting the 16S rRNA encoding gene present in metagenomic DNA were produced using a barcoded primer set adapted for the Illumina HiSeq2000 and MiSeq (Caporaso et al., 2012). DNA sequence data were then generated using Illumina paired‐end sequencing at the Environmental Sample Preparation and Sequencing Facility (ESPSF) at Argonne National Laboratory. Specifically, the V4 region of the 16S rRNA gene (515F‐806R) was PCR amplified with region‐specific primers that include sequencer adapter sequences used in the Illumina flow cell (Caporaso et al., 2011, 2012). The forward amplification primer also contains a twelve base barcode sequence that supports pooling of up to 2,167 different samples in each lane (Caporaso et al., 2011, 2012). Each 25 µl PCR reaction contained 9.5 µl of MO BIO PCR Water (Certified DNA‐Free), 12.5 µl of QuantaBio's AccuStart II PCR ToughMix (2× concentration, 1× final), 1 µl Golay barcode tagged Forward Primer (5 µM concentration, 200 pM final), 1 µl Reverse Primer (5 µM concentration, 200 pM final), and 1 µl of template DNA. The conditions for PCR were as follows: 94°C for 3 min to denature the DNA, with 35 cycles at 94°C for 45 s, 50°C for 60 s, and 72°C for 90 s, with a final extension of 10 min at 72°C to ensure complete amplification. Amplicons were then quantified using PicoGreen (Invitrogen) and a plate reader (Infinite 200 PRO, Tecan). Once quantified, volumes of each of the products were pooled into a single tube so that each amplicon is represented in equimolar amounts. This pool was then cleaned up using AMPure XP Beads (Beckman Coulter) and then quantified using a fluorometer (Qubit, Invitrogen). After quantification, the molarity of the pool was determined and diluted down to 2 nM, denatured, and then diluted to a final concentration of 6.75 p.m. with a 10% PhiX spike for sequencing on the Illumina MiSeq. Amplicons were sequenced on a 151 bp × 12 bp × 151 bp MiSeq run using customized sequencing primers and procedures (Caporaso et al., 2012).

#### 2.3.2 ITS sequencing for fungal community

Genomic DNA was amplified using an internal transcribed spacer (ITS) barcoded primer set, adapted for the Illumina HiSeq2000 and MiSeq. These primers were designed by Kabir Peay's lab at Stanford University (Smith & Peay, 2014). The reverse amplification primer also contained a 12 base barcode sequence that supports pooling of up to 2,167 different samples in each lane (Caporaso et al., 2011, 2012). Each 25 µl PCR reaction contained 9.5 µl of MO BIO PCR Water (Certified DNA‐Free), 12.5 µl of QuantaBio's AccuStart II PCR ToughMix (2× concentration, 1× final), 1 µl Golay barcode tagged Forward Primer (5 µM concentration, 200 pM final), 1 µl Reverse Primer (5 µM concentration, 200 pM final), and 1 µl of template DNA. The conditions for PCR were also as follows: 94°C for 3 min to denature the DNA, with 35 cycles at 94°C for 45 s, 50°C for 60 s, and 72°C for 90 s, with a final extension of 10 min at 72°C to ensure complete amplification. Amplicons were quantified using PicoGreen (Invitrogen) and a plate reader. Once quantified, different volumes of each of the products were pooled into a single tube so that each amplicon was equally represented. This pool was then cleaned up using AMPure XP Beads (Beckman Coulter) and then quantified using a fluorometer (Qubit, Invitrogen). After quantification, the molarity of the pool was determined and diluted down to 2 nM, denatured, and then diluted to a final concentration of 6.75 p.m. with a 10% PhiX spike for 2 × 251 bp sequencing on the Illumina MiSeq.

#### 2.3.3 Bioinformatics

Sequence data were processed and analyzed using the plugin‐based microbiome bioinformatics framework QIIME 2 (Bolyen et al., 2019). DADA2 (Callahan et al., 2016) was used (via the q2‐dada2 QIIME 2 plugin) to quality filter the sequence data, removing PhiX, chimeric, and erroneous reads. The 16S rRNA gene sequences were too short to overlap paired‐end reads after denoising, so only forward reads were used for downstream analysis, trimmed at 151 nt. The plugin q2‐cutadapt (Martin, 2011) was used to trim primers and adapters from the ITS sequence data prior to paired‐end DADA2 denoising. To construct a phylogenetic tree, 16S rRNA gene sequence variants were inserted into the Greengenes version 13_8 reference phylogeny (McDonald et al., 2012) using the q2‐fragment‐insertion plugin (Janssen et al., 2018). Taxonomy was assigned to sequence variants using q2‐feature‐classifier (Bokulich, Kaehler, et al., 2018) with the classify‐sklearn method against the Greengenes 16S rRNA reference database 13_8 release (McDonald et al., 2012) or against the UNITE ITS reference database (Nilsson et al., 2018).

QIIME 2’s q2‐diversity plugin was used to estimate alpha diversity (within‐sample diversity) using the following metrics: richness (as observed sequence variants), Shannon diversity and evenness, and Phylogenetic diversity (Faith, 1992). Microbiome beta diversity (between‐sample diversity) was estimated in QIIME 2 using weighted and unweighted UniFrac distance (Lozupone & Knight, 2005). Feature tables were evenly subsampled at 4,000 sequences per sample (for ITS data) or 15,618 sequences per sample (16S rRNA gene data) prior to alpha or beta diversity analyses. Appropriate rarefaction levels were chosen using alpha rarefaction to determine where richness estimates converge toward the asymptote, indicating that sampling richness has been saturated; in the case of the 16S rRNA gene data, 15,618 sequences were chosen as the sequencing depth of the lowest‐coverage sample, well beyond the minimum appropriate rarefaction depth. Two‐way ANOVA tests were performed (using the q2‐longitudinal plugin, Bokulich, Dillon, Zhang, et al., 2018) to test whether alpha diversity estimates differed between plant species and blocks, or between tomato yield, tomato biomass, or phylogenetic distance to tomato. Two‐way permutational multivariate analysis of variance tests (Anderson, 2001; as implemented in the adonis method in the vegan R package (Oksanen et al., 2018), wrapped via the q2‐diversity plugin) were performed to test whether beta diversity estimates partitioned by block, plant species, tomato yield, or phylogenetic distance to tomato.

Supervised learning methods are increasingly being used to characterize and differentiate microbial communities across samples (Bokulich, Dillon, Bolyen, et al., 2018). For example, supervised learning can be used to identify patterns in microbiome data that relate to different groups of samples (e.g., across experimental treatments or environmental gradients). Supervised learning was performed in q2‐sample‐classifier (Bokulich, Dillon, Bolyen, et al., 2018) via fivefold nested cross‐validation (classify‐samples‐ncv method), using random forest classification or regression models (Breiman, 2001) grown with 100 trees. Supervised learning was performed to determine how accurately sample groups can be distinguished based on taxonomic profiles and phylogenetic distance to tomato using microbial abundance data as features; 16S rRNA gene and fungal ITS sequence data tables were merged and used to train learning models.

DEseq2 (Love, Huber, & Anders, 2014) was used to test whether microbial species abundances were differentially abundant between blocks, plant species, and based on phylogenetic distance to tomato.

## 3 RESULTS

### 3.1 Phylogenetic relatedness does not affect tomato yield

**Figure 1:**
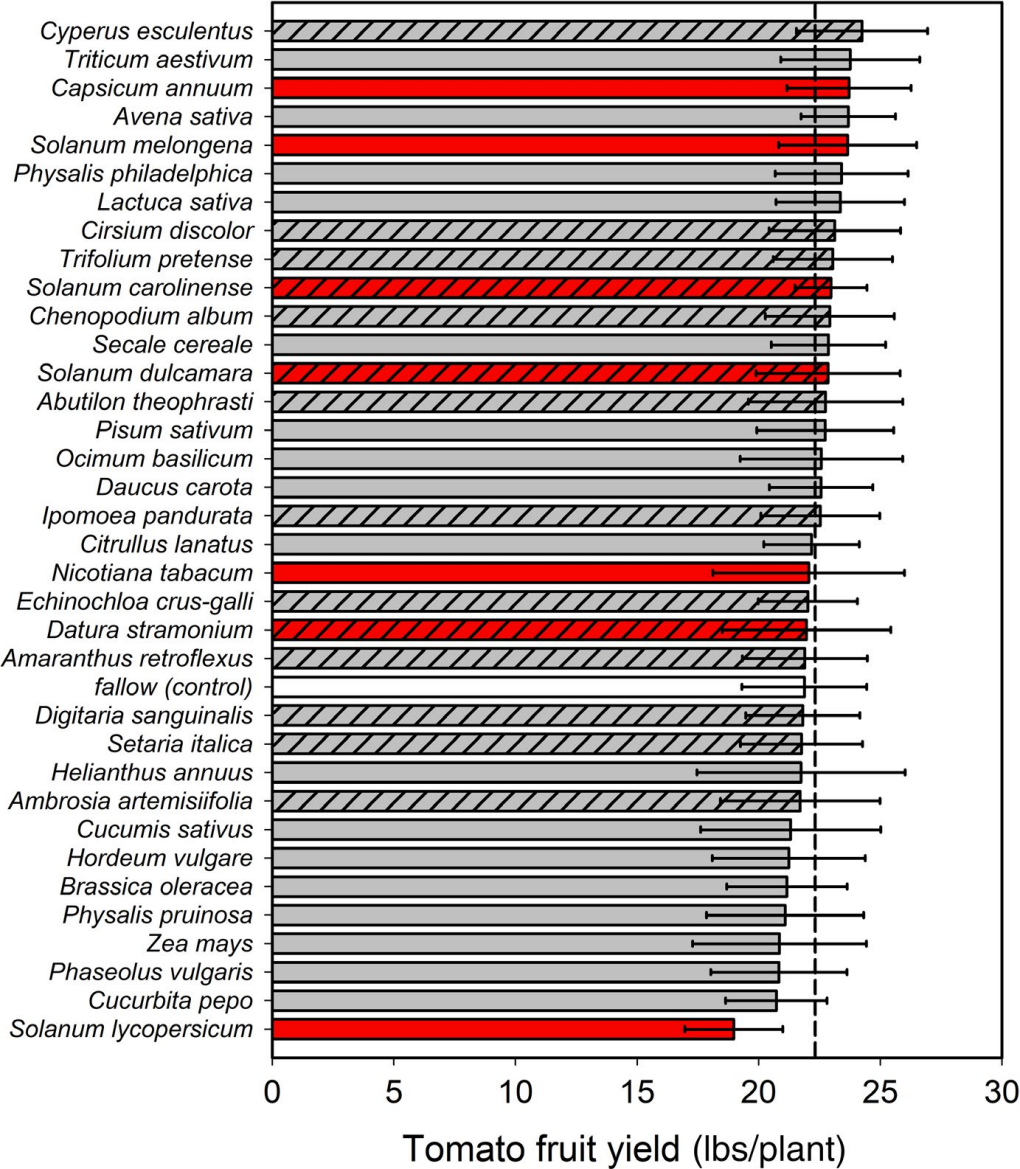
Impact of identity of the conditioning plant species in year 1 (*y*‐axis) on tomato yield (mean ± 95% CI) in year 2 (*x*‐axis). Vertical dashed line represents the global mean across all plots (= 22.27); thus, 95% CIs that do not bracket this line over‐ or under‐perform relative to the community average. Red bars represent plants that are closely related to tomato, that is, in the family Solanaceae, while the white bar denotes the plant‐free fallow control. Hatched bars separate weed species from crops (unhatched)

**Figure 2:**
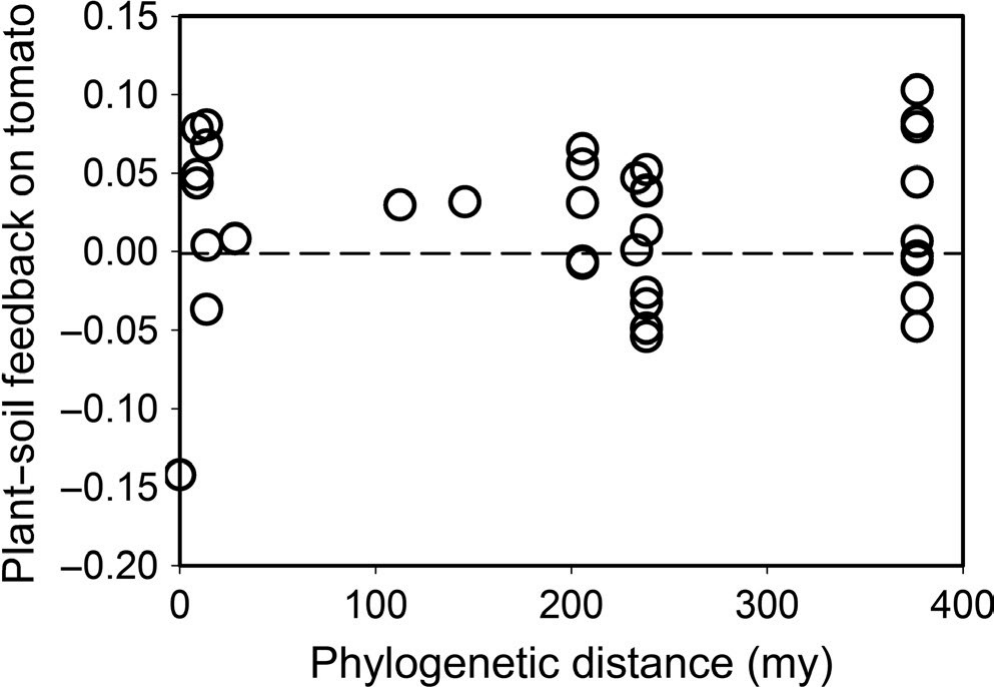
Relationship between phylogenetic distance separating tomato from the species conditioning the soil in year 1 and plant–soil feedback on tomato yield in year 2. Each point is the mean across all plot replicates for the 35 plant species tested. Horizontal dashed line denotes an effect size of zero where the conditioning plant species had no impact on tomato growth relative to the control. Effect size was calculated as ln(species/control)

Of the 35 conditioning species cultivated in year one, tomato was the only species whose soil legacy impacted tomato yield in year two; that is, 95% CI does not bracket the community mean in Figure 1. However, this negative feedback did not extend past the species level to other plants in the genus *Solanum* or family Solanaceae (see other red bars in Figure 1 aside from *S. lycopersicium*). As a result, there was no overall relationship between the phylogenetic distance separating tomato from the rotation species in year one and tomato yield in year two (*t*(1) = 0.20, *p* = .8429; Figure 2).

These outcomes correspond with categorical comparisons using major taxonomic thresholds. Tomatoes growing in self (tomato) plots had 15.3% lower yield than tomatoes growing in non‐self (i.e., all other nontomato plant species) plots (*p* < .001). However, neither genus (*p* = .724) nor family (*p* = .996) had any predictive explanatory power on tomato growth. In fact, yield was nearly identical when comparing across these broader taxonomic groups. Similarly, tomato yield was the same in fallow control plots compared with any of the treatments receiving plants (*p* = .643).

Importantly, none of the soil nutrients showed strong evidence for explaining species‐level differences in crop performance, namely the lower yields in tomato plots. When comparing the nutritional profiles of tomato versus nontomato soils, none were significant at the Bonferroni‐corrected *p* = .003 level. The only mineral trending in this direction (*p* = .030) was potassium, which was lower in tomato (322.25 ppm ± 27.67 SE) than nontomato soils (394.28 ppm ± 6.21 SE).

### 3.2 Plant species imprint unique phylogenetic signatures on the soil microbiome

**Figure 3:**
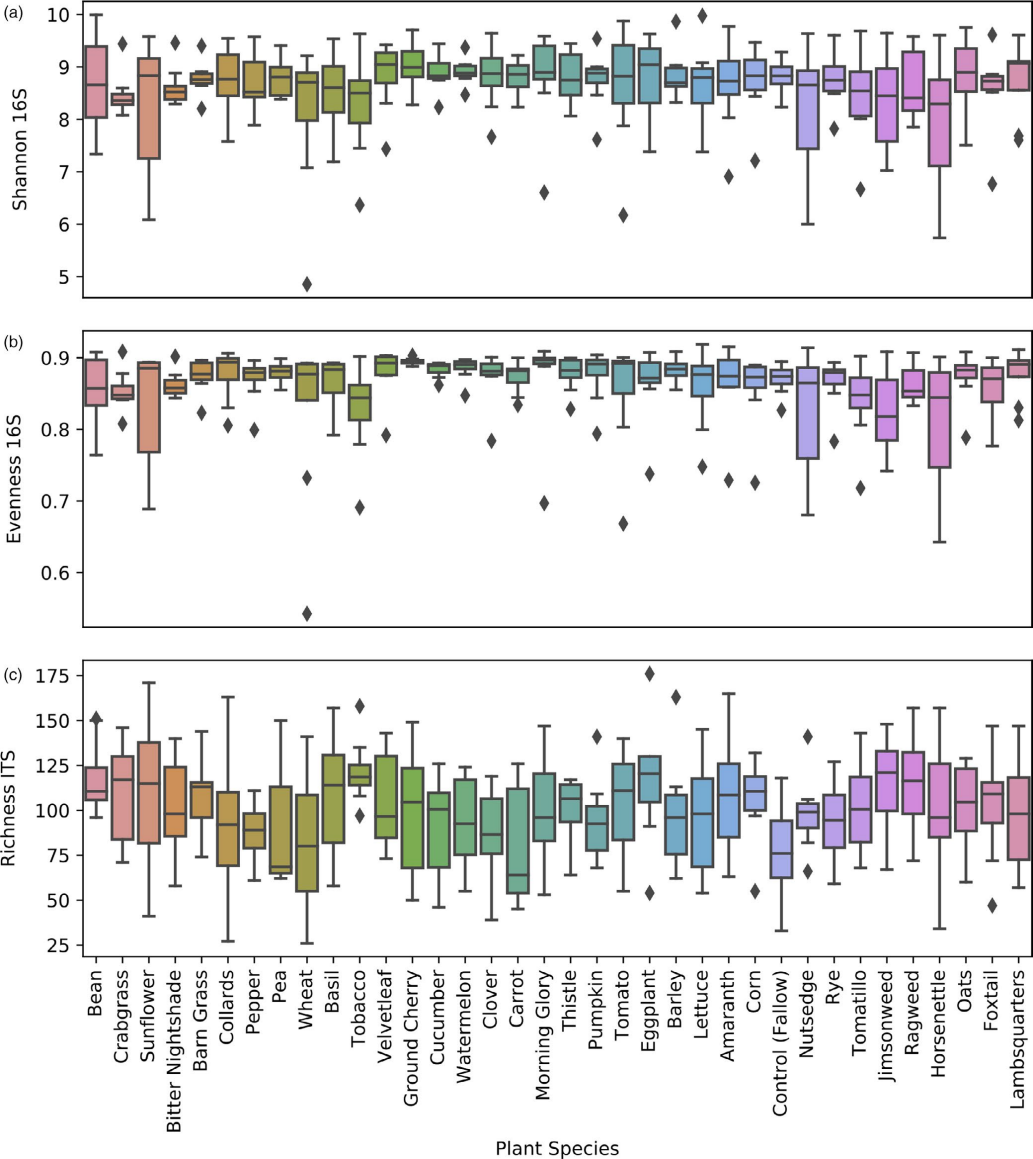
Alpha diversity by plant species. (a) Bacterial 16S rRNA gene Shannon diversity after even rarefaction at 15,618 sequences per sample. (b) Bacterial 16S rRNA gene evenness after even rarefaction at 15,618 sequences per sample. (c) Fungal ITS richness (observed sequence variants) after even rarefaction at 4,000 sequences per sample. This figure displays all alpha diversity metrics that were significantly associated with plant species (Table 1)

Both bacterial and fungal alpha diversity (i.e., within‐sample biodiversity) were impacted by block, which was highly significant for all response variables, and secondarily by plant species, which affected some but not all responses (Table 2). Because of the overriding statistical influence of block effects on bacterial and fungal profiles, this spatial factor is accounted for in all subsequent measurements. Bacterial Shannon H (Figure 3a; *p* = .038) and evenness (Figure 3b; *p* = .003) differed between plant species, but neither richness nor phylogenetic diversity were significantly affected (*p* > .05). Fungal richness, on the other hand, was different between plant species (Figure 3c; *p* = .006), but Shannon H and evenness were not (*p* > .05).

**Table 2:** ANOVA results for plant species and block effects on alpha diversity of bacteria (16S) and fungi (ITS) in field‐collected soil

Marker	Metric^a^	Factor	*df*	*F*	*p*
16S	Richness	Plant species	35	0.88	.663
		Block	7	66.72	<.001
	Evenness	Plant species	35	1.89	.003
		Block	7	18.88	<.001
	Shannon	Plant species	35	1.52	.038
		Block	7	34.02	<.001
	Faith's PD	Plant species	35	0.92	.602
		Block	7	43.46	<.001
ITS	Richness	Plant species	35	1.80	.006
		Block	7	32.85	<.001
	Evenness	Plant species	35	1.04	.410
		Block	7	39.64	<.001
	Shannon	Plant species	35	1.20	.212
		Block	7	44.63	<.001

^a^ Each marker × metric combination represents a separate ANOVA test (metric ~ plant species + block) (total *N* = 6 tests).

Planting history also exhibited a substantial impact on both bacterial and fungal beta diversity (i.e., between‐sample dissimilarity). Plant species had a significant effect on all beta diversity metrics for both bacterial and fungal communities (PERMANOVA *p* < .001; Table 3) and accounted for between 15% and 23% of the variation in beta diversity. Block also significantly impacted beta diversity (*p* < .001), accounting for between 6% and 25% of the variation, which was less explanatory power than plant species for all metrics except for fungal Bray–Curtis dissimilarity.

**Table 3:** PERMANOVA results for plant species and block effects on beta diversity of bacteria (16S) and fungi (ITS) in field‐collected soil

Marker	Metric^a^	Factor	*df*	*R* ^2^	*F*	*p*
16S	Bray–Curtis	Plant Species	35	.20	1.96	<.001
		Block	7	.10	4.69	<.001
		Residuals	244	.71		
		Total	286	1.00		
	Jaccard	Plant Species	35	.15	1.33	<.001
		Block	7	.06	2.68	<.001
		Residuals	244	.79		
		Total	286	1.00		
	Unifrac	Plant Species	35	.15	1.29	<.001
		Block	7	.06	2.63	<.001
		Residuals	244	.79		
		Total	286	1.00		
	wUnifrac	Plant Species	35	.23	2.53	<.001
		Block	7	.13	7.41	<.001
		Residuals	244	.63		
		Total	286	1.00		
ITS	Bray–Curtis	Plant Species	35	.15	1.73	<.001
		Block	7	.25	14.26	<.001
		Residuals	243	.60		
		Total	285	1.00		
	Jaccard	Plant Species	35	.15	1.29	<.001
		Block	7	.06	2.82	<.001
		Residuals	243	.79		
		Total	285	1.00		

^a^ Each marker × metric combination represents a separate PERMANOVA test (distance ~ plant species + block) (total *N* = 6 tests).

**Figure 4:**
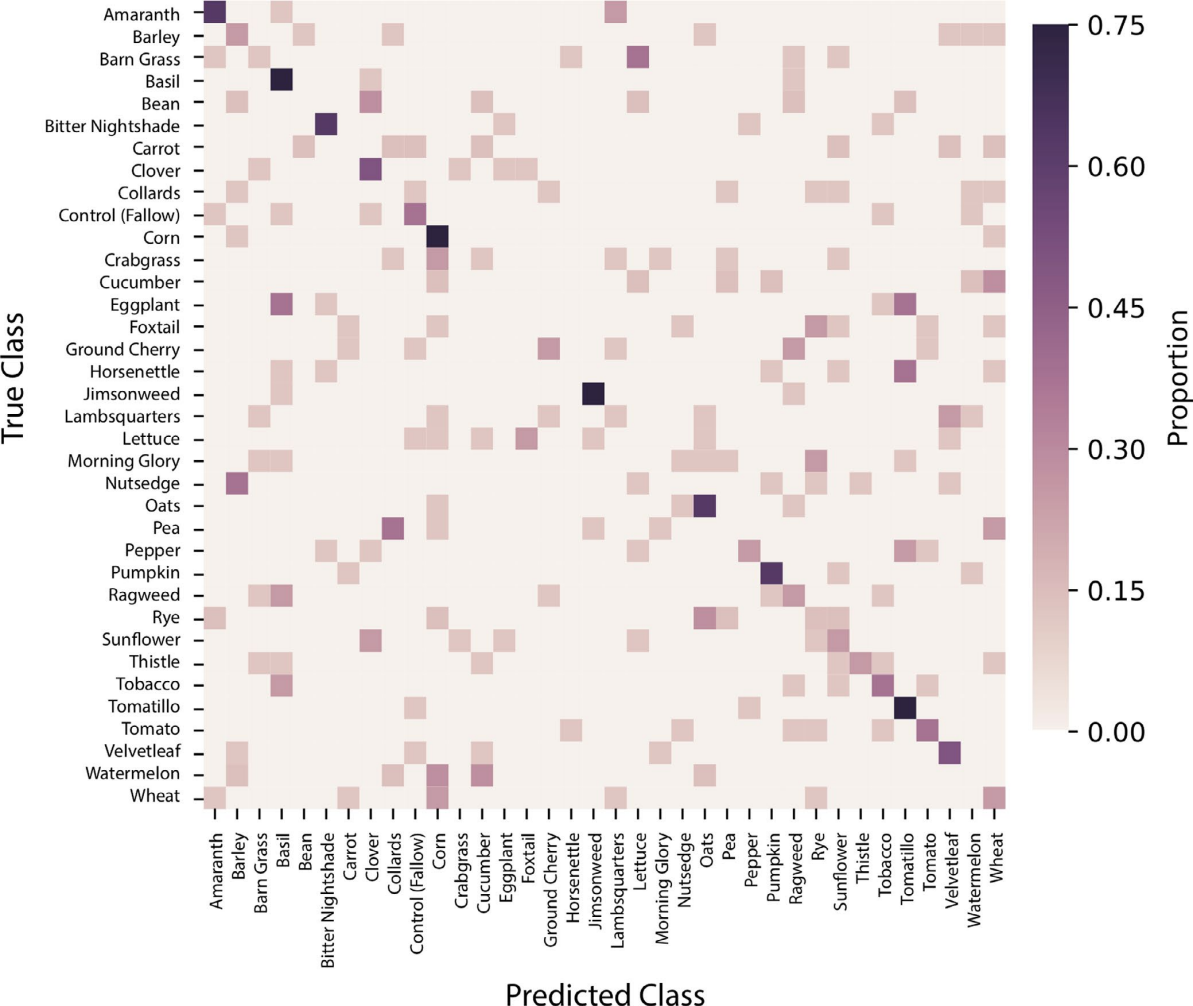
Random forest classification correctly identifies soil planting history 27.6% of the time via fivefold cross‐validation. Confusion matrix shows the predicted planting history of each sample, as the proportion of times that samples in each class were predicted to belong to each possible class

**Figure 5:**
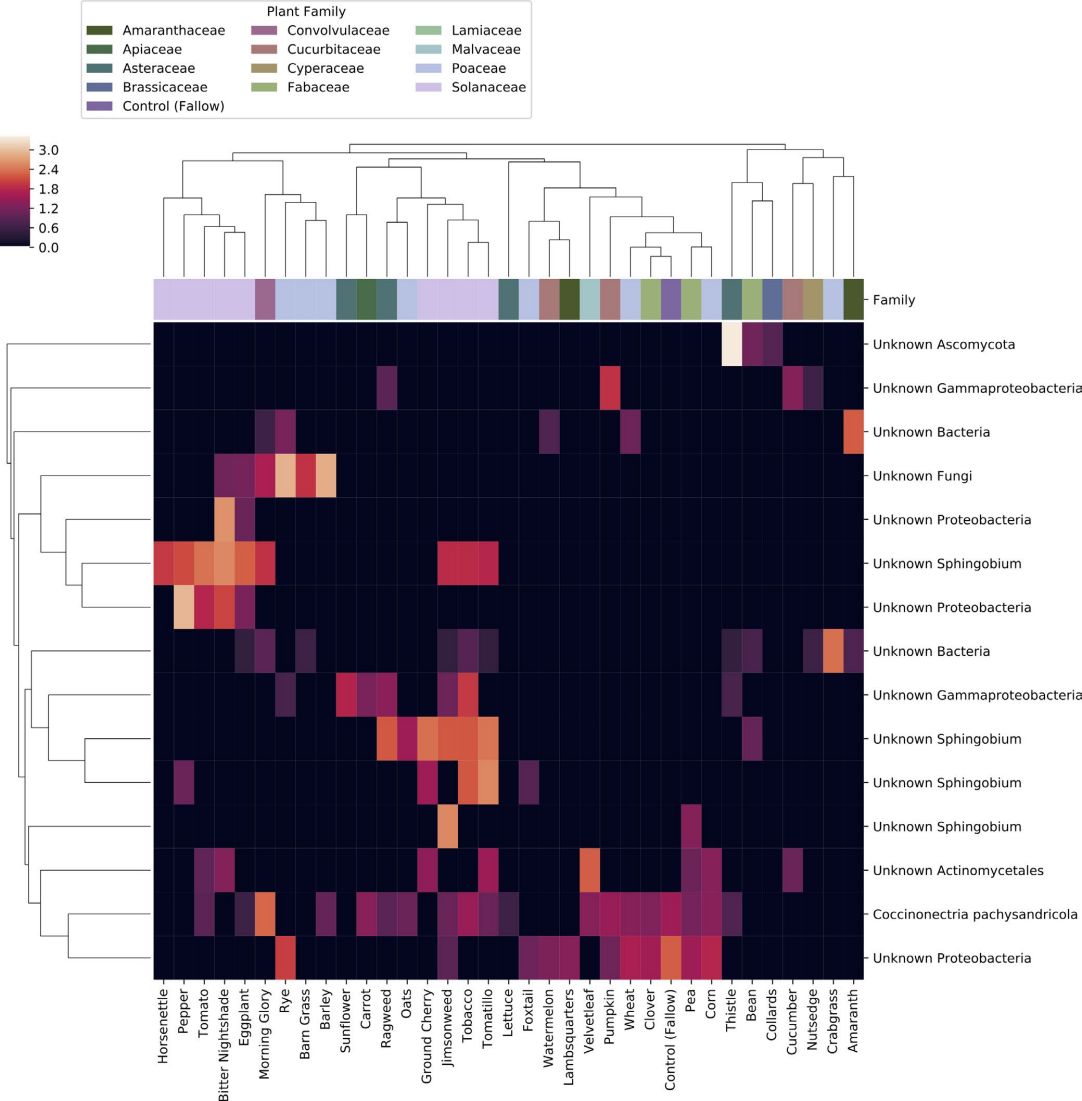
Random forest models identify microbial features predictive of planting history. The top 15 most predictive bacterial and fungal features are shown (minimum importance score 0.010), and the heatmap displays their normalized average relative abundances within each plant species. Samples and features are hierarchically clustered by UPGMA of pairwise Bray–Curtis dissimilarities

Next, we identified features that differentiate plant species to determine how planting history alters the relative abundance of specific microorganisms in the soil. To achieve this, we trained random forest classifiers on combined bacterial and fungal feature tables and subsequently inspected the resulting models to determine which features were most relevant for differentiating previous plant species. Random forest classifiers, using the combined bacterial and fungal community, could accurately predict the previous plant species grown in each soil plot 27.6% of the time (Figure 4), a nearly 10‐fold improvement over the baseline accuracy rate of 2.8% (the accuracy rate that would be achieved by assigning all samples to the most common class). The predictive features are primarily bacterial and include several *Sphingobium* species that are most abundant in soils previously planted with Solanaceae, indicating that this may be a group of bacteria that is enriched by that plant family (Figure 5). Interestingly, different *Sphingobium* species are associated with two different clusters of Solanaceae, separating *Solanum* and *Capsicum* sp. from other Solanaceae regardless of domestication status (crop versus weed), suggesting genus‐specific associations among Solanaceae and *Sphingobium* groups.

**Figure 6:**
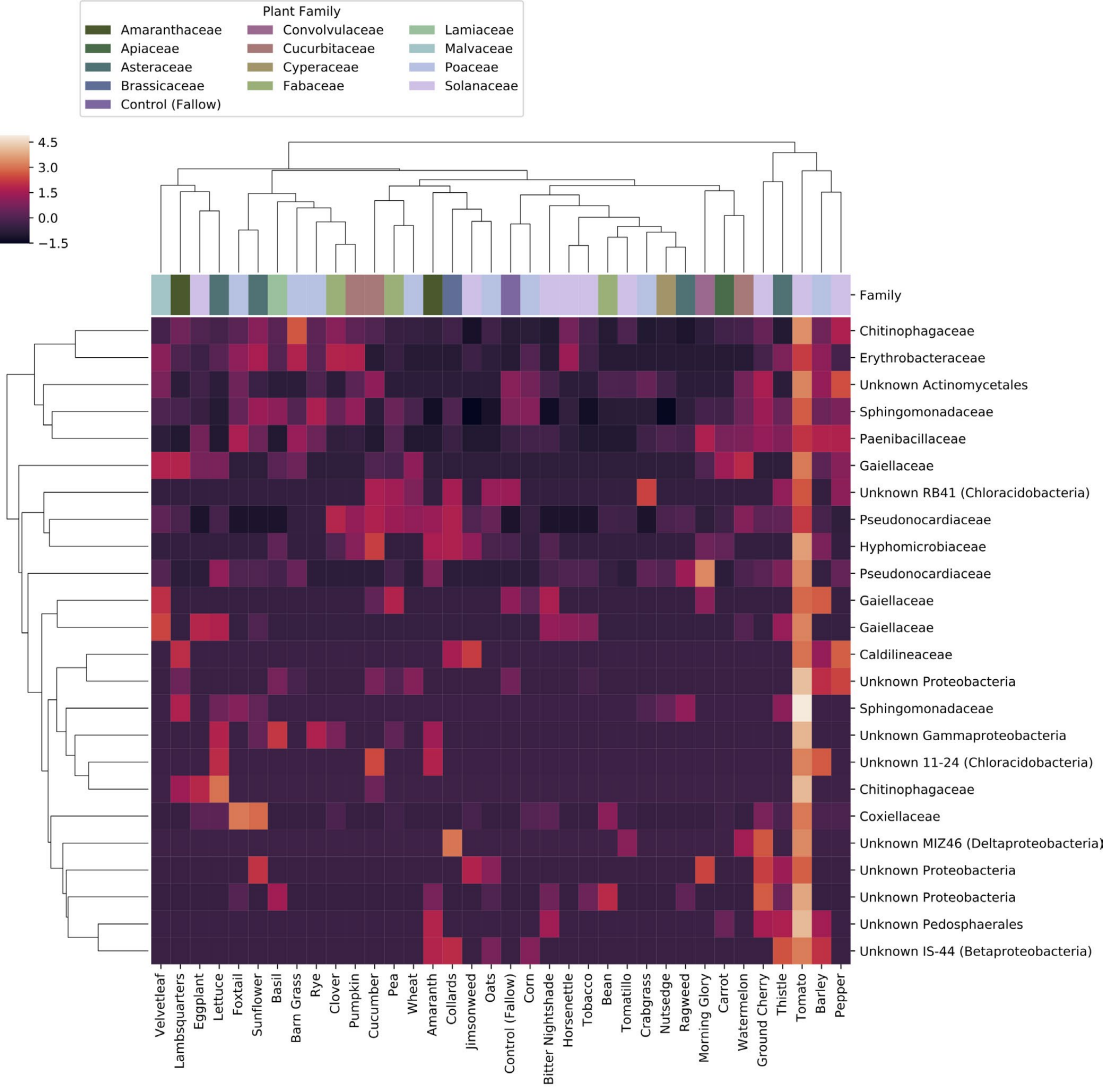
Bacterial features most abundant in tomato soils. The heatmap shows the normalized relative abundance of 24 features identified as being differentially abundant among plant species and block, and most abundant in tomato soils. Values shown on the heatmap represent the mean of each feature averaged across each plant species (*N* = 8 per species). Column margin colors indicate the family affiliation of each plant species represented in that column. Samples and features are hierarchically clustered by UPGMA of pairwise Bray–Curtis dissimilarities

**Figure 7:**
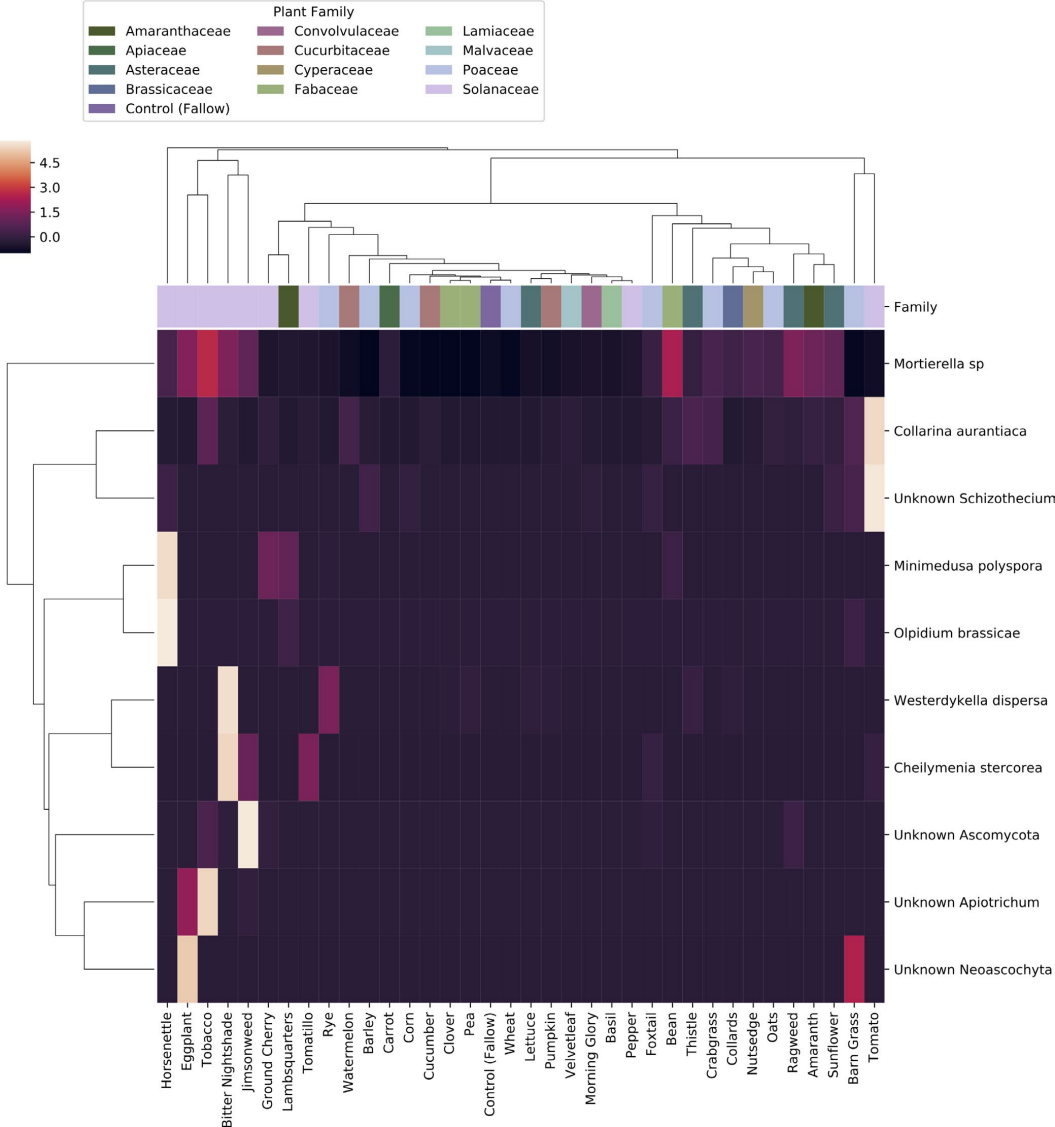
Fungal features most abundant in Solanaceae soils. The heatmap shows the normalized relative abundance of 10 features identified as being differentially abundant among plant species and block, and most abundant in Solanaceae soils. Values shown on the heatmap represent the mean of each feature averaged across each plant species (*N* = 8 per species). Column margin colors indicate the family affiliation of each plant species represented in that column. Samples and features are hierarchically clustered by UPGMA of pairwise Bray–Curtis dissimilarities

DeSeq2 was also used to identify bacterial and fungal features that were differentially abundant between plant species and block. A total of 1,394 bacterial features (239 most abundant in Solanaceae, 24 most abundant in tomato; Figure 6) and 213 fungal features (10 most abundant in Solanaceae, 2 in tomato; Figure 7) were differentially abundant (FDR‐corrected *p* < .01). Among the differentially abundant bacteria were 27 sequence variants classified as Sphingomonadaceae, including one *Sphingobium*, supporting the links between this group and plant species.

PERMANOVA tests indicate that both bacterial and fungal beta diversity are associated with phylogenetic distance to tomato (*p* < .05), indicating an association between plant phylogeny and microbiome composition; however, all distance metrics yielded low *R* ^2^ values (≤.01), indicating that plant evolutionary history explains little variation in beta diversity. Similarly, Mantel tests indicated significant correlation between host plant phylogeny (not distance to tomato) and bacterial and fungal pairwise distances (*p* < .05), with the exception of bacterial weighted UniFrac.

**Figure 8:**
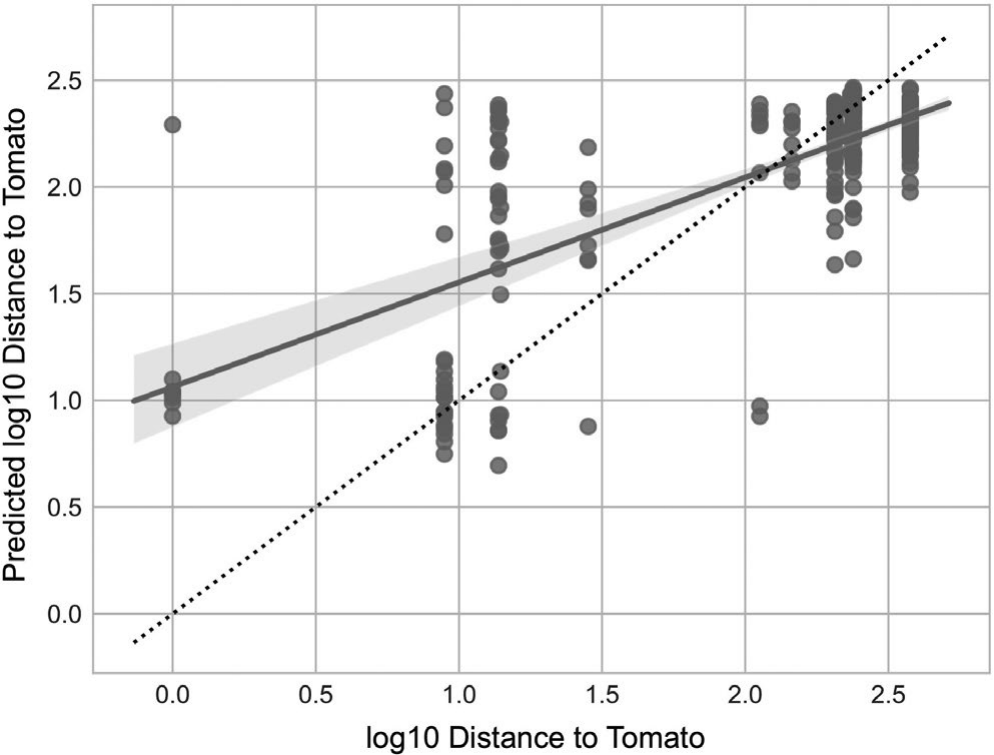
Random forest regression between microbiota and genetic distance to tomato suggests that phylogenetically related plants harbor similar soil microbial communities. Fivefold cross‐validation was used to train/predict log10 genetic distance to tomato as a function of soil microbiota composition across all plant species samples collected in this study. The scatterplot shows the relationship between true and predicted genetic distance. The gray line indicates the linear regression trend line between true and predicted values (Pearson *R* ^2^ = .52, *p* < .001; mean squared error = 0.21). The dotted line marks the ideal 1:1 relationship between true and predicted values

Last, we identified microbial features that were predictive of the host plant species’ genetic distance to tomato. Random forest regression predicted log 10 distance to tomato with moderate accuracy via fivefold cross‐validation (Figure 8; Pearson *R* ^2^ = .52, *p* < .001; mean squared error = 0.21). Again, we see several *Sphingobium* species among the top 16 most predictive features (minimum 0.03 importance score), associated with various Solanaceae species (Figure S1); these include many of the same *Sphingobium* sequence variants that predict plant species (Figure 5). These results indicate that a number of microbial species (primarily bacteria) are enriched in soils following Solanaceae cultivation, and their abundance is weakly predictive of the genetic distance between that plant and tomato, indicating species‐ and family‐specific microbial associations.

## 4 DISCUSSION

Given the purported relationship between planting history and soil microbiome composition, we hypothesized that microbial community structure is linked to the evolutionary history of the host plant. Such a relationship would justify phylogenetically informed crop rotation practices, under the assumption that microbes associated with plant species represent host‐specific pathogenic or performance‐reducing taxa. Overall, we found mixed support for this hypothesis. Although phylogeny predicted some of the variation underlying plant legacy on soil microbial communities, phylogenetic relationships were entirely uninformative past the species level for predicting differences in tomato performance. Thus, we conclude that plant phylogeny is moderately important in structuring the microbiome of agricultural soils, but has no value in forecasting changes to yield in multi‐species cropping systems. Notably, these general conclusions mirror those from a recent study conducted on short‐term vegetative growth of potted, greenhouse tomatoes using the same experimental plant community (Ingerslew & Kaplan, 2018).

Several putative factors could lead to a “phylogenetic breakdown” across levels whereby the same evolutionary pattern is not passed along from microbes to plants (i.e., if phylogeny structures microbes and microbes mediate plant health, then why is phylogeny unrelated to tomato performance?). First, microbes may not be the primary mechanism responsible for changes to crop yield. While it is widely assumed that soil microbes influence plant health and performance, plant–soil feedbacks are also driven by variation in growth‐limiting micronutrients (van der Putten, Bradford, Brinkman, Voorde, & Veen, 2016). We consider this explanation less likely, compared with natural ecosystems, since fields were fertilized, which should dilute nutritional differences across plant species treatments. Yet, phosphorus levels were 18% lower in tomato soils compared with all other plots, which could serve as a factor contributing to lower yields in self versus non‐self treatments. Phosphorus deficiency is known to reduce tomato growth and reproduction (Biddinger, Liu, Joly, & Raghothama, 1998; Menary & Staden, 1976).

Second, the large degree of functional redundancy among microbial taxa in the rhizosphere could result in detectable changes to the taxonomic composition of the microbiome, but without ultimately affecting the overall impact of that soil on plant health (Berendsen, Pieterse, & Bakker, 2012; Vandenkoornhuyse, Quaiser, Duhamel, Van, & Dufresne, 2015). This is particularly relevant given the relatively high level of resolution that recent advances in sequencing and statistical technologies allow for discerning even subtle shifts to microbial community structure (nontargeted identification of microbial genera and species, Bokulich, Kaehler, et al., 2018). Similarly, the compositional changes to the microbiome could involve taxa that are weakly tied to plant health, rather than pathogens or mutualists. Of the microbial groups correlated with tomato in this study, none are well‐documented pathogens. For example, bacteria in the genus *Sphingobium* were closely affiliated with tomato and the Solanaceae family in general. This association has been reported from other studies of the tomato rhizosphere (Kim, Dungan, Kwon, & Weon, 2006; Kwak et al., 2018; Lee et al., 2016; Renaut, Masse, Norrie, Blal, & Hijri, 2019) and even tomato flowers (Kwon, Lee, Kim, Jeon, & Kwak, 2018), suggesting that *Sphingobium* are tightly linked to aboveground and belowground tomato growth or reproduction. Unfortunately, the functional roles these bacteria play are unclear. Some have suggested they degrade secondary metabolites from root exudates or cells in the soil (Pascual et al., 2016). They also controlled lettuce corky root disease when experimentally tested, demonstrating a potential role in disease suppression (van Bruggen, Francis, & Jochimsen, 2014). Targeted genomics of *Sphingobium* sp. detected in our study, as well as shotgun metagenome and metatranscriptome experiments, will elucidate the functional roles of these species and communities (respectively), as well as their interactions with the host plants.

The paucity of fungi among the predictive features in our statistical models suggests that plant species exert a stronger effect on bacterial composition or that bacteria are more likely to form species‐specific relationships. Alternatively, ITS primer bias (Bokulich & Mills, 2013) could lead to selective amplification of fungal taxa in the soil. Multi‐locus amplification and/or shotgun metagenome sequencing could be used in future studies to provide a more complete view of fungal diversity, yielding greater insight into plant–fungal associations in the soil. PCR cycle count is known to introduce subtle biases in amplicon sequencing experiments (Sze & Schloss, 2019) and could be an additional factor that reduced the apparent differentiation of fungal profiles between closely related crop species in this study.

Regardless of the specific microbial groups driving phylogenetic patterns, a few notable outcomes emerged from the overall analysis. For one, the random forest regression revealed that predictive models were far more accurate at identifying distant plant relatives based on the soil microbiome than close relatives (i.e., compare solid versus dashed lines in Figure 8). The model consistently predicted that other solanaceous plants were more distant relatives than their true evolutionary history indicates. In addition, the regression illustrates that the model is more consistent in assigning an evolutionary classification to close and distant relatives; those plants intermediate in their relatedness to tomato were highly variable. In other words, the spread among the datapoints along the y‐axis is more pronounced in the middle—0.75 to 1.75—distances compared with close and distant relatives where the predicted values tend to cluster around a central point. This outcome is further supported by random forest classification where certain plant species imprint highly distinct microbial signatures (see dark purple squares along the diagonal in Figure 4), whereas others leave virtually no discernible legacy. To our knowledge, the reasons underlying why some plants generate long‐term, species‐specific legacies on the soil microbiome and others do not are unknown.

As a whole, our data indicate that phylogenetic relatedness should not be used as a proxy for plant complementarity in multi‐species crop rotations. The only rule consistent with current dogma is that consecutive plantings of the same species in shared soil should be avoided whenever possible. This means that crops should be rotated with a different species, but the identity of that rotation partner over time is not necessarily contingent on whether they are congeners or come from opposite ends of the plant kingdom. We suspect that certain crop pairings are beneficial and synergize based on somewhat idiosyncratic aspects of how those species respond to the others’ legacy. An important caveat to these conclusions is that our experimental design, due to the large number of species treatments, was established using a plant–soil feedback framework based on how plants respond from one year to the next. True rotation studies, however, implement long‐term rotations that simulate how farms produce crops in reality. If several nontomato *Solanum* species were rotated over a 5‐ or 10‐year period, this could lead to the development of soil‐borne diseases that we did not observe in a simple, two‐year feedback study. Similarly, low‐input (e.g., organic) systems with different abiotic and biotic pressures could change the relationship between phylogeny and yield. Our experiment was also conducted with a single crop and soil type; more studies are needed using a wider diversity of crop species and locations before broader conclusions can be drawn. Nevertheless, these data clearly illustrate the limitations to applied phylogenetics in agriculture and suggest that future cropping system studies test rotations that vary relatedness as part of their experimental design.

## CONFLICT OF INTEREST

None declared.

## Supporting information

Fig S1

## Data Availability

Raw sequence data are accessible in the Qiita repository (ID 12546; Gonzalez et al., 2018).
