# Supplementary figures and images for: Phylogenetic farming: Can evolutionary history predict crop rotation via the soil microbiome?
Source: Evol Appl. 2020 Apr 22;13(8):1984–99. doi: 10.1111/eva.12956 (PMC7463318; doi:10.1111/eva.12956)

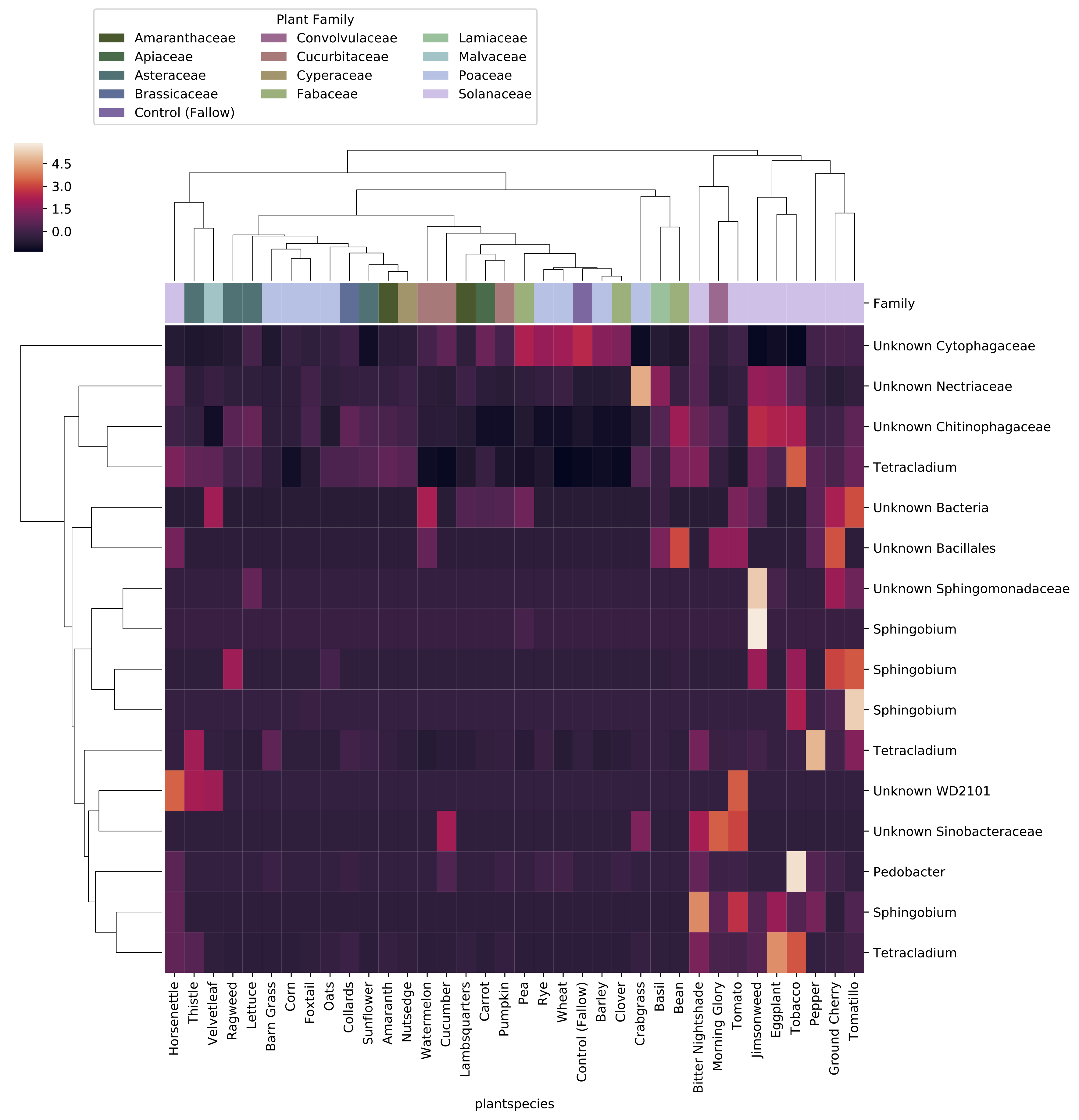

Supplement: Supplementary file 1 — Fig S1 [file EVA-13-1984-s001.tiff]
